# Detection of new drivers of frequent B-cell lymphoid neoplasms using an integrated analysis of whole genomes

**DOI:** 10.1371/journal.pone.0248886

**Published:** 2021-05-04

**Authors:** Adrián Mosquera Orgueira, Roi Ferreiro Ferro, José Ángel Díaz Arias, Carlos Aliste Santos, Beatriz Antelo Rodríguez, Laura Bao Pérez, Natalia Alonso Vence, Ággeles Bendaña López, Aitor Abuin Blanco, Paula Melero Valentín, And´res Peleteiro Raindo, Miguel Cid López, Manuel Mateo Pérez Encinas, Marta Sonia González Pérez, Máximo Francisco Fraga Rodríguez, José Luis Bello López

**Affiliations:** 1 Health Research Institute of Santiago de Compostela (IDIS), Santiago de Compostela, Galicia, Spain; 2 Department of Hematology, Complexo Hospitalario Universitario de Santiago de Compostela (CHUS), SERGAS, Santiago de Compostela, Galicia, Spain; 3 University of Santiago de Compostela, Santiago de Compostela, Galicia, Spain; 4 Department of Pathology, Complexo Hospitalario Universitario de Santiago de Compostela (CHUS), SERGAS, Santiago de Compostela, Galicia, Spain; German Cancer Research Center (DKFZ), GERMANY

## Abstract

B-cell lymphoproliferative disorders exhibit a diverse spectrum of diagnostic entities with heterogeneous behaviour. Multiple efforts have focused on the determination of the genomic drivers of B-cell lymphoma subtypes. In the meantime, the aggregation of diverse tumors in pan-cancer genomic studies has become a useful tool to detect new driver genes, while enabling the comparison of mutational patterns across tumors. Here we present an integrated analysis of 354 B-cell lymphoid disorders. 112 recurrently mutated genes were discovered, of which *KMT2D*, *CREBBP*, *IGLL5* and *BCL2* were the most frequent, and 31 genes were putative new drivers. Mutations in *CREBBP*, *TNFRSF14* and *KMT2D* predominated in follicular lymphoma, whereas those in *BTG2*, *HTA-A* and *PIM1* were more frequent in diffuse large B-cell lymphoma. Additionally, we discovered 31 significantly mutated protein networks, reinforcing the role of genes such as *CREBBP*, *EEF1A1*, *STAT6*, *GNA13* and *TP53*, but also pointing towards a myriad of infrequent players in lymphomagenesis. Finally, we report aberrant expression of oncogenes and tumor suppressors associated with novel noncoding mutations (*DTX1* and *S1PR2*), and new recurrent copy number aberrations affecting immune check-point regulators (*CD83*, *PVR*) and B-cell specific genes (*TNFRSF13C*). Our analysis expands the number of mutational drivers of B-cell lymphoid neoplasms, and identifies several differential somatic events between disease subtypes.

## Introduction

B-cell lymphoid neoplasms are the most frequent hematological tumors, and they exhibit a diverse spectrum of entities with heterogeneous clinical behaviour. B-cell lymphoid neoplasms are classically classified in either aggressive lymphomas (DLBCL, Burkitt lymphoma, grade III follicular lymphoma and mantle cell lymphomas), or indolent lymphomas (chronic lymphocytic leukemia (CLL), grade I/II follicular lymphoma, marginal zone lymphoma, lymphoplasmacytic lymphoma…). By frequency, diffuse large B-cell lymphoma (DLBCL) is the most frequent lymphoid neoplasm, accounting for 25% of all cases of non-Hodgkin lymphoma (NHL), closely followed by CLL (19% of NHLs) and follicular lymphoma (12% of NHLs) [1].

Next-generation sequencing (NGS) technologies have tried to deconvolute the genomic complexity of lymphoid tumors. This information has led to an improved classification of lymphoid neoplasms, mainly thanks to the characterization of the biological heterogeneity within lymphoma subtypes. A good example is that of the gene expression-based classification of DLBCL in two different clinico-biological groups by its cell-of-origin status: either germinal center B cell-like or activated B cell-like [2]. Various groups have also identified new DLBCL subtypes based on their mutational profiles, also observing a correlation between some of these mutational patterns with cell-of-origin status [3, 4]. In the same line, cumulative evidence indicates that co-occurring mutations are drivers of treatment refractoriness and clonal evolution in follicular lymphoma [5, 6]. In the same line, significant advances in the deconvolution of the genomic landscapes of both CLL and Burkitt lymphoma have been made in the past years [7–11], providing new disease-specific drivers, hypermutation events and predictors of adverse outcome. Some key findings include the predominance of *ID3* mutations in Burkitt lymphoma, but not in other *IGH-MYC* rearranged lymphomas [12], as well as the role of aberrant somatic hypermutation (aSHM) in Epstein-Barr positive Burkitt Lymphomas [11]. Such somatic hypermutation in the *IGHV* locus of CLL tumors is also important, as it defines two important types of leukemia which exhibit broadly different clinical and mutational backgrounds [8]. Additionally, some of the mutational drivers of CLL are also important mediators of drug resistance, such as in the case of rituximab-resistance observed in *NOTCH1*-mutated CLLs [13].

An additional line of complexity is conformed by the limited comprehension of the contribution of regulatory mutations to the pathogenesis of cancer. Existing research points towards the deregulation of important driver genes by noncoding mutations in lymphomas. For example, *Batmanov et al*. (2017) discovered regulatory mutations that control *BCL2* and *BCL6* expression in follicular lymphoma [14]; *Arthur et al*. (2018) identified aberrant expression of *NFKBIZ* in DLBCL caused by functional noncoding mutations in the 3’ untranslated region of the gene [15], and *Puente et al*. (2015) characterized enhancer mutations that deregulate *PAX5* expression in CLL [8]. Considering the extensive heterogeneity of these disorders, we anticipate that the analysis of larger and diverse patient cohorts will enable the identification of new regulatory driver regions of B-cell tumors.

Although increasing NGS data in cancer is available, the detection of driver mutations continues to be a bottleneck in the development of this technology. Differences in clonality, sample purity, sequencing coverage and quality are challenging for most variant callers. These are addressed using different methods, leading to remarkable disparities in results between algorithms [16]. *Hoffman et al*. [17] compared 10 variant callers on simulated data, reporting considerable differences in sensitivity and precision depending on coverage and variant allele frequency. Concordantly, *Cai et al*. [18] analyzed a set of cancer samples with four different algorithms and observed that only 20.7% of variants were detected by ≥2 callers. Therefore, numerous pathogenic variants in large sequencing projects could have passed unnoticed. Furthermore, cancer genomes suffer from the *“long tail”* phenomenon, whereby a few driver genes are recurrently mutated and most mutations are distributed across a vast number of genes [19]. Both the enhanced statistical power and the capacity to analyze divergent molecular mechanisms across tumor types are the main reasons that motivate the increasingly common aggregation of tumors in pan-cancer genomic studies [20–22].

In this report we present an integrated genomic analysis of diverse mature B-cell lymphoid neoplasms using whole genome sequencing (WGS) data produced by the *International Cancer Genome Consortium* (ICGC) [23]. Our results expand the catalog of B-cell lymphoma driver genes, identify novel putative drivers based on functionally connected subnetworks and characterize new structural aberrations and regulatory mutations that modify the expression of several oncogenes and tumor suppressors.

## Methods

### 1. Data source and analysis

WGS data from the *CLLE-ES* and *MALY-DE* projects produced by the *ICGC* were analyzed. This cohort included 132 CLL, 36 Burkitt lymphoma, 85 DLBCL, 97 follicular lymphoma and 4 unspecified B-cell lymphoma cases. RNAseq expression data for a subgroup of the samples was also available.

Tumor-normal matched whole genomes were processed using the bcbio-nextgen pipeline, which provides best practices for NGS data analysis [24]. GRCh37 was used as the reference genome. Low complexity regions, areas with abnormally high coverage, sequences with single nucleotide stretches >50bp and loci with alternative or unplaced contigs in the reference genome were not analyzed. Some polymorphic regions in noncoding regions are prone to be classified as mutation hotspots due to artifacts or biases in the sequencing process (mainly in low coverage regions), and suspicious elements were manually discarded from downstream analysis. Single nucleotide and indel mutation detection was performed with *vardict-java* version 1.5.8 [25], *varscan* version 2.4.3 [26], *mutect2* implemented in GATK version 3.8 [27] and *freebayes* version 1.1.0.46 [28] using default bcbio-nextgen parameters. Events with a minimum sequencing depth (DP) of 10 and a genotype quality (GQ) of 20 Phred in both tumor and normal samples were selected. A mutation was called when detected by ≥2 callers. Mutations were annotated to the *1000G* [29], *gnomAD* [30] and *ExAc* [31] databases. Tumor mutations reported as polymorphisms with a minimum allele frequency > 0.001 in any population were discarded. For copy number aberration (CNA) detections, the *CNVkit* version 0.9.6a0 [32] algorithm was used (ploidy-adjusted and with default parameters). We initially used the circular binary segmentation algorithm, observing important hypersegmentation that could lead to an increase in false positives. Therefore, we finally used the *HaarSeg* segmentation method, retrieving a vast majority of cases with segment counts in the range of 100–200. Events detected in centromeric and telomeric regions were discarded. Similarly, we also removed events within low 100bp-read mappability regions according to *UCSC* tracks.

### 2. Detection of mutation drivers in coding regions

Three methods were used to detect driver genes using nonsynonymous coding mutation data: *MutSigCV* [33], *dNdScv* [34] and *OncodriveFML* [35]. MutSigCV version 1.3.5 and dNdSCV were run with default parameters. OncodriveFML was run using CADD 1.3 scores. Significance threshold was set at FDR of 10% for all methods.

*Hierarchical HotNet* [36] was used to infer networks of functionally connected mutated genes. The following protein-protein interaction networks were used: *Hint+Hi2012*, *Irefindex9* and *Multinet*. Mutation frequency and log-transformed *MutSigCV* p-values were used as input scores. Heat scores were permuted 100 times for each network. Hierarchies were constructed and processed with default parameters. The deviation of observed dendrogram distribution from the random expectation at different similarity thresholds was calculated, and significance threshold was set to p-value <0.05. Finally, a consensus network (G_2_) was created from the resulting significant subnetworks.

### 3. Noncoding region annotation and mutation enrichment analysis

Annotations corresponding to promoter regions, 5’UTR, 3’UTR and lincRNAs were retrieved from *Genecode* version 18 [37]. Enhancer regions were obtained from the *GeneHancer* database [38], and those supported by two or more sources of evidence (“elite” enhancers) were selected. Transcription start sites (TSS) were defined as the 100bp-region upstream of the point of transcription. Regulatory regions within telomeric and centromeric positions were discarded.

*LARVA* [39] was used to identify areas with evidence of positive selection of mutations. *LARVA* models the mutation counts of each target region as a β-binomial distribution in order to handle overdispersion. *LARVA* also includes replication timing information in order to estimate local mutation rate, and provides a β-binomial distribution adjusted for replication timing which is used to compute p-values. Significance threshold was set to FDR<10%. As we used *LARVA* including tumor classification data, the mutation background estimation was calculated for each tumor subtype.

Regions targeted by aSHM were retrieved from literature analysis [40–42], and were used to annotate the list of significantly mutated noncoding regions identified by *LARVA*. In the case of genes without annotation, we used Signal [43] to test for local enrichments in the mutational signature of aSHM.

### 4. Recurrent focal CNA detection

*Gistic2*.*0* [44] was used to identify recurrent CNA. Focal CNA were defined as those spanning a maximum of 25% of an arm’s length. Deletions were called in regions with tumor/normal log ratios < -0.3, and amplifications in regions with ratios >0.3. Evenly spaced pseudomarkers were automatically created by the algorithm, and regions were considered only if they spanned 10 or more pseudomarkers. Sex chromosomes were not analyzed. Arm-level peel off was enabled, and residual q-values were calculated after removing segments shared with higher peaks. Significance threshold was set to FDR of 10%.

### 5. Gene expression analysis and association with CNA and regulatory mutations

RNA-seq data from tumor samples were transformed to FPKM counts and then rank normalized. The Wilcoxon-Rank sum test was used to detect changes in gene expression between mutated and wild-type cases. Changes in expression of the nearest gene were analyzed. When multiple regulatory regions mapped the same gene, p-values were adjusted for multiple testing using the FDR method (significance threshold of 5%). In the case of significant associations, we used non-linear Kernel regression adjusted for disease subtype in order to rule out independence from diagnostic subtype [45]. In the case of CNA, association with expression of the affected genes was analyzed using Pearson’s correlation. P-values were adjusted for multiple testing using the FDR method (significance threshold of 5%).

### 6. Differential distribution of somatic events and pathways analysis

Differential mutation analysis between the different disease subtypes was performed using Fisher’s exact test (significance FDR threshold of 5%).

*WebGestalt* [46] was used to analyze enrichment of gene networks in biological pathways. The *KEGG* database was used as reference, and a significance threshold of FDR 5% was chosen.

## Results

### 1. Mutation landscape of B-cell lymphoid malignancies

5,743,241 mutations were detected in 354 B-cell malignancy samples. A minor proportion of these affected protein-coding regions (1.34%), of which 71.64% were non-synonymous. Among these, missense mutations predominated (87.64%), followed by nonsense mutations (6.79%) and splice-site mutations (2.76%). The vast majority of mutations were either intergenic (40.28%) or intronic (45.25%). Mutation rate in the cohort was 2.51 mutations/Mb. This mutation rate was different for the different B-cell neoplasms. Mutation rate was 0.48 mutations/Mb in CLL, 0.75 in Burkitt Lymphoma, 3.14 mutations/Mb in follicular lymphoma and 5.69 mutations/Mb in DLBCL.

### 2. Identification of significantly mutated genes

**Fig 1:**
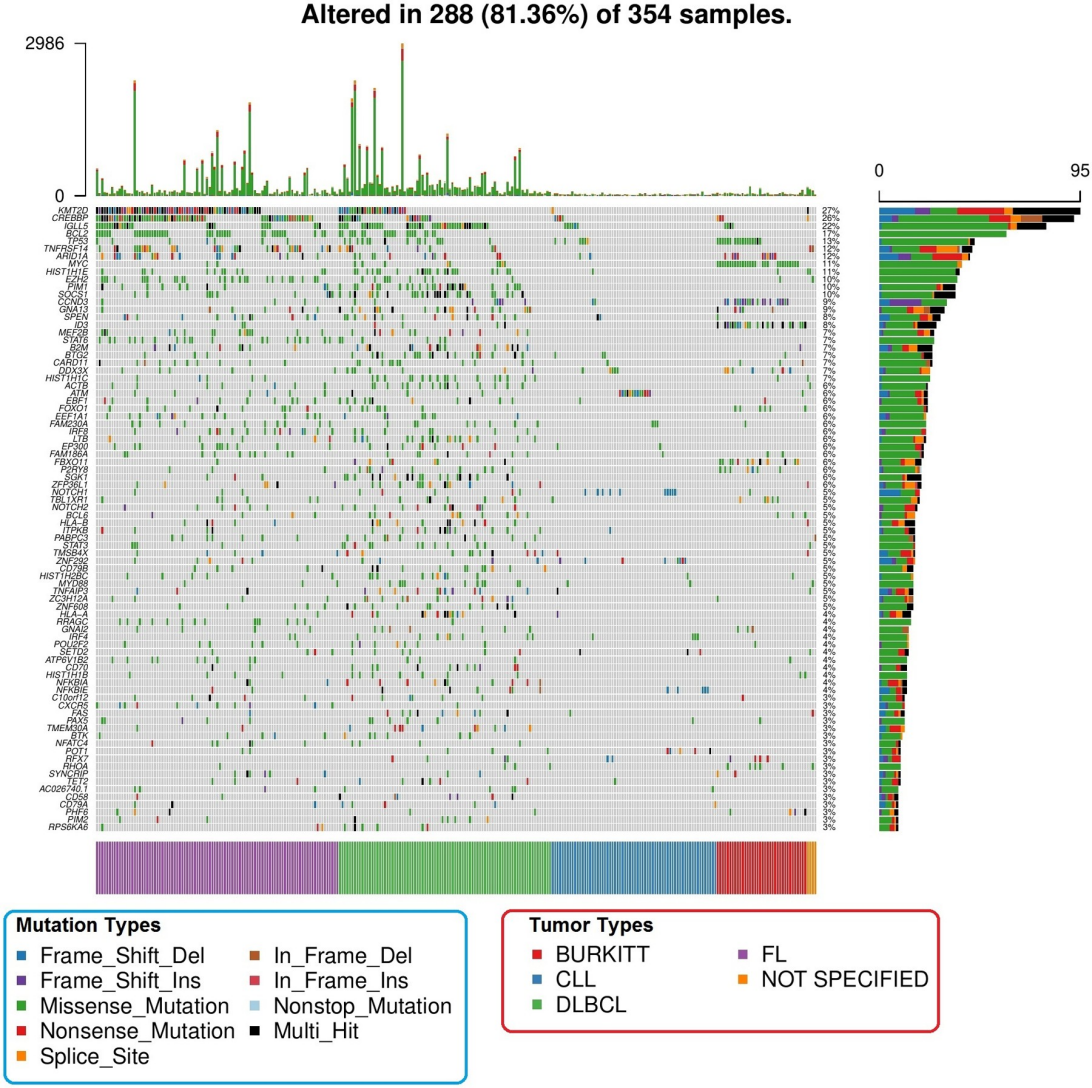
Representation of the most frequent drivers (frequency > 3%) across all the samples. Tumor subtypes are color-coded in the lower bar, mutation distribution is represented in the right-side bars, and per sample mutation number is represented in the top bars.

**Fig 2:**
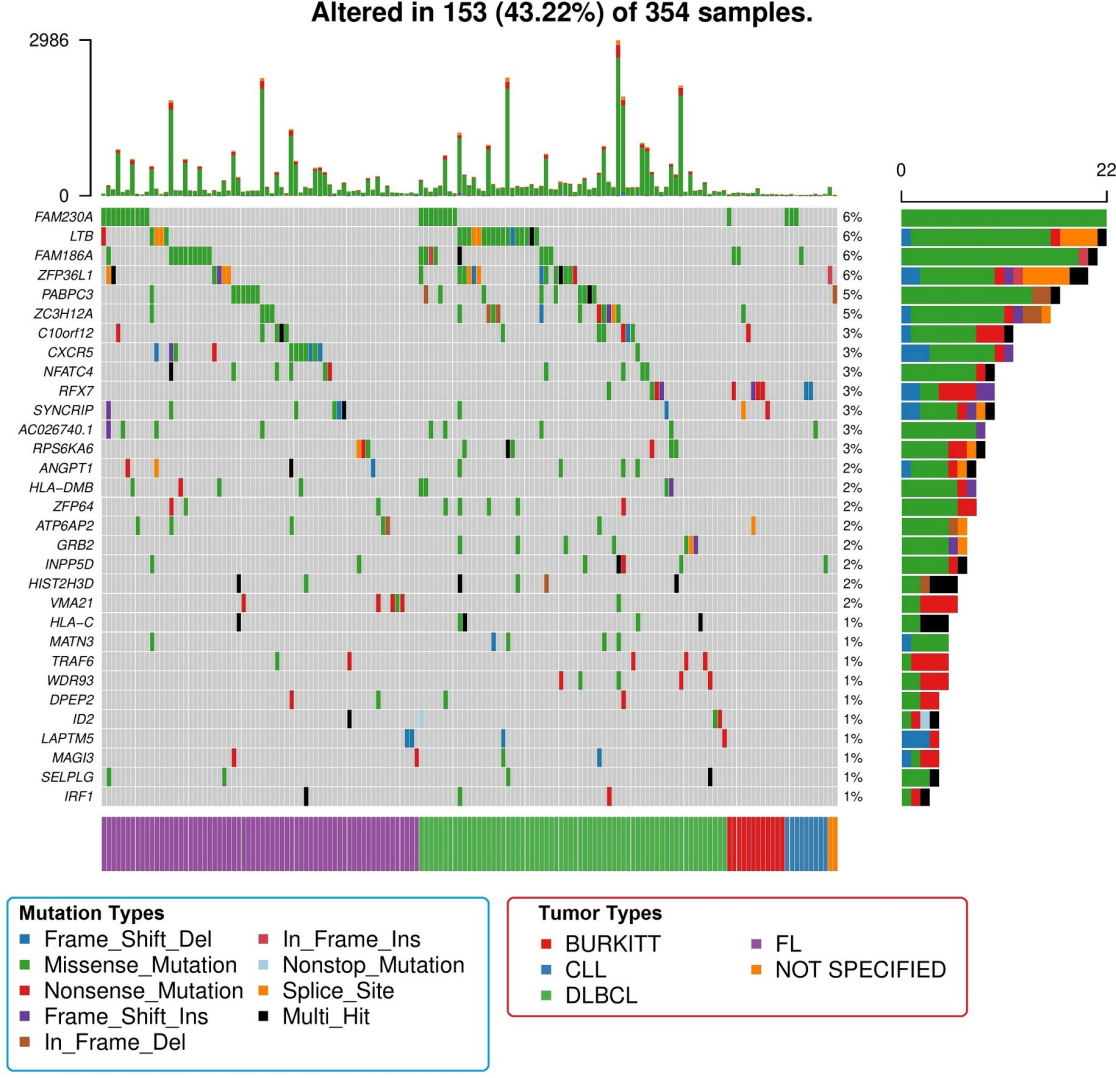
Representation of the new drivers discovered in this analysis. Image details are identical to those of the previous image.

*dNdSCV*, *OncodriveFML* and *MutSigCV* detected 88, 52 and 46 recurrently mutated genes, respectively (FDR <10%) (**S1–S3 Tables**). Overall, 112 genes were detected as significantly mutated by any of the methods (**S4 Table**). The most frequently mutated were *KMT2D* (27%), *CREBBP* (26%), *IGLL5* (22%), *BCL2* (17%), *TP53* (13%), *ARID1A* (12%) and *TNFRSF14* (12%) (**Fig 1**). 31 genes were not previously described as recurrently mutated in any of the lymphoid malignancies analyzed (**Fig 2**), and these affected 43.22% of patients. The most frequent were *FAM230A* (6%), *LTB* (6%), *FAM186A* (6%), *ZFP36L1* (6%), *PABPC3* (5%) and *ZC3H12A* (5%). Among these, only *LTB* and *ZFP36L1* have been previously described as targets of aSHM.

Missense mutations were the most frequent at the exome level, but some of these novel drivers were predominantly affected by other types of mutations. Nonsense mutations were more frequent in *MAGI3*, *RFX7*, *TRAF6*, *VMA21* and *WDR93*. The genes *HIST2H3D* and *HLA-C* were prone to suffer multiple types of mutations in the same patient, whereas frameshift deletions predominated in *LAPTM5*. Finally, the following genes were targeted by multiple mutation types: *ANGPT1*, *ID2*, *IRF1*, *MAGI3*, *SYNCRIP* and *ZFP36L1*.

### 3. Detection of low-frequency drivers by functionally altered subnetwork analysis

**Fig 3:**
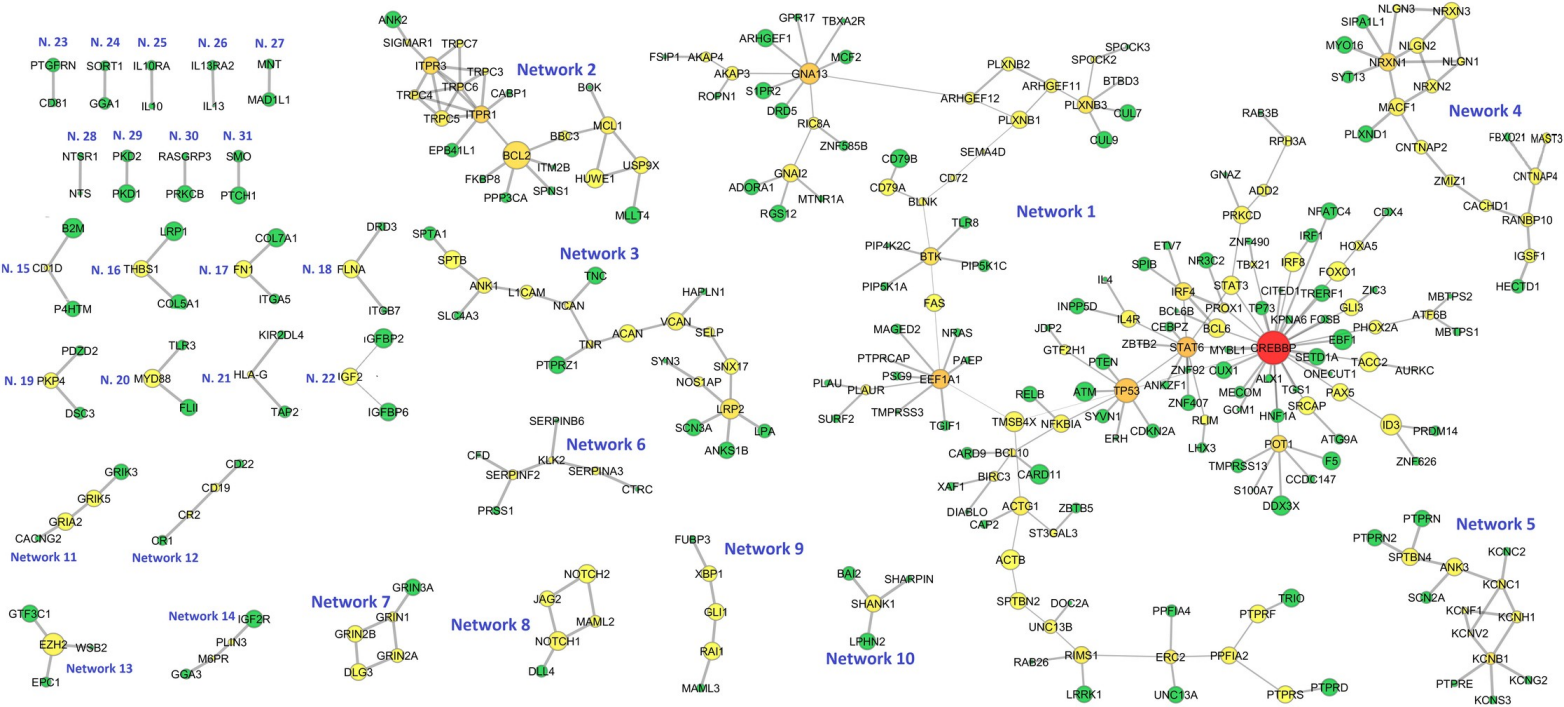
Representation of all significantly mutated protein subnetworks according to Hierarchical HotNet results. Node size is proportional to mutation frequency, node color is proportional to node degree (red: higher degree values, green: lower degree values) and edge width is proportional to betweenness centrality.

31 significantly mutated protein subnetworks were detected, involving 313 different genes (**Fig 3 and S5 Table**). 8 networks were mutated in >10% patients. The widest network (Network 1) was composed of 153 genes, among which *CREBBP*, *EEF1A1*, *GNA13*, *STAT6* and *TP53* were the main hubs. This network was enriched in pathways such as *“B cell receptor signalling pathway”*, *“Hepatitis B”* and *“NFKB signalling pathway”* (**S6 Table and S1 Fig**). The second widest network (Network 2) was composed of 22 genes centered around *BCL2*. As expected, this network was notoriously enriched in *“Apoptosis”* pathway genes (**S6 Table and S2 Fig**). The third and fourth biggest networks (Networks 3 and 4) were composed of 20 genes each. A significant enrichment in *“Cell Adhesion Molecules”* pathway genes characterized Network 4 (FDR 2.65 x 10^−9^).

Multiple of the remaining subnetworks include genes involved in oncogenesis. For example, Network 8 is composed of 5 genes of the *“Notch signalling”* pathway. Network 12 contains B-cell markers (*CD19* and *CD22*) as well as complement proteins (*CR1*/*CD35* and *CR2*/*CD21*). Network 20 contains genes of the toll-like receptor pathway (*MYD88* and *TLR3*), and Networks 25 and 26 are integrated by interleukins and their respective receptors (*IL10* and *IL10RA*; *IL13* and *ILR13RA2*). Furthermore, other less explored routes in lymphomagenesis emerged as significantly mutated. These included cell signalling proteins (*MAML3*, *PRKCB*, *PTPRN2* and *RASGRP3*), gene expression and cell-cycle regulators (*GLI1*, *MAD1L1*, *MNT*, *PDZD2*, *XBP1*), surface receptors and signal transduction proteins (*CD81*, *DRD3*, *GRIN1*, *GRIA2*, *GRIK3*, *LPHN2*, *PTCH1*, *PTPRE*, *PTPRN*, *SMO* …), angiogenesis regulators (*BAI2*), ion transport genes (*KCNB1*, *KCNC1*, *KCNC2*, *KCNG2*, *PKD1*, *PKD2* …), cytoskeleton and cell adhesion molecules (*ANK3*, *COL5A1*, *COL7A1*, *DSC3*, *ITGB7*, *ITGA5*, *FLNA*, *FN1*, *PKP4*, *SHANK1*, *SPTBN4*), growth factors (*IGF2* and *IGF2R*), immunity genes (*B2M*, *CD1D*, *HLA-G*, *KIR2DL4*, *TAP2*,), vesicle trafficking proteins (*GGA1*, *GGA3*, *M6PR*, *PLIN3* and *SORT1*) and extracellular enzymes (*CFD*, *CTRC*, *KLK2*, *SERPINA3*, *SERPINB6*, *SERPINF2*).

### 4. Regions enriched in non-coding DNA mutations

Significant enrichments in 180 regulatory elements mapping to 73 different genes were discovered. These involved 54 promoters, 53 UTRs, 33 enhancers, 21 TSS and 19 lincRNAs (**S7 Table**). 122 of these regions overlapped genes targeted by aSHM in lymphomas, and signature analysis indicated that another 4 regions were also likely targets of aSHM. On the contrary, 54 regions affecting 25 different genes were not affected by aSHM by signature analysis. Pathway analysis revealed significant overlaps between these genes and the following pathways: *“Transcriptional misregulation in cancer”* (q-value 8.55 x 10^−3^), *“Pathways in cancer”* (q-value 8.55 x 10^−3^), *“MicroRNAs in cancer”* (q-value 1.37 x 10^−2^), *“JAK-STAT signaling pathway”* (q-value 1.41 x 10^−2^), *“Toxoplasmosis”* (q-value 1.82 x 10^−2^) and *“Apoptosis”* (q-value 3.53 x 10^−2^).

**Fig 4:**
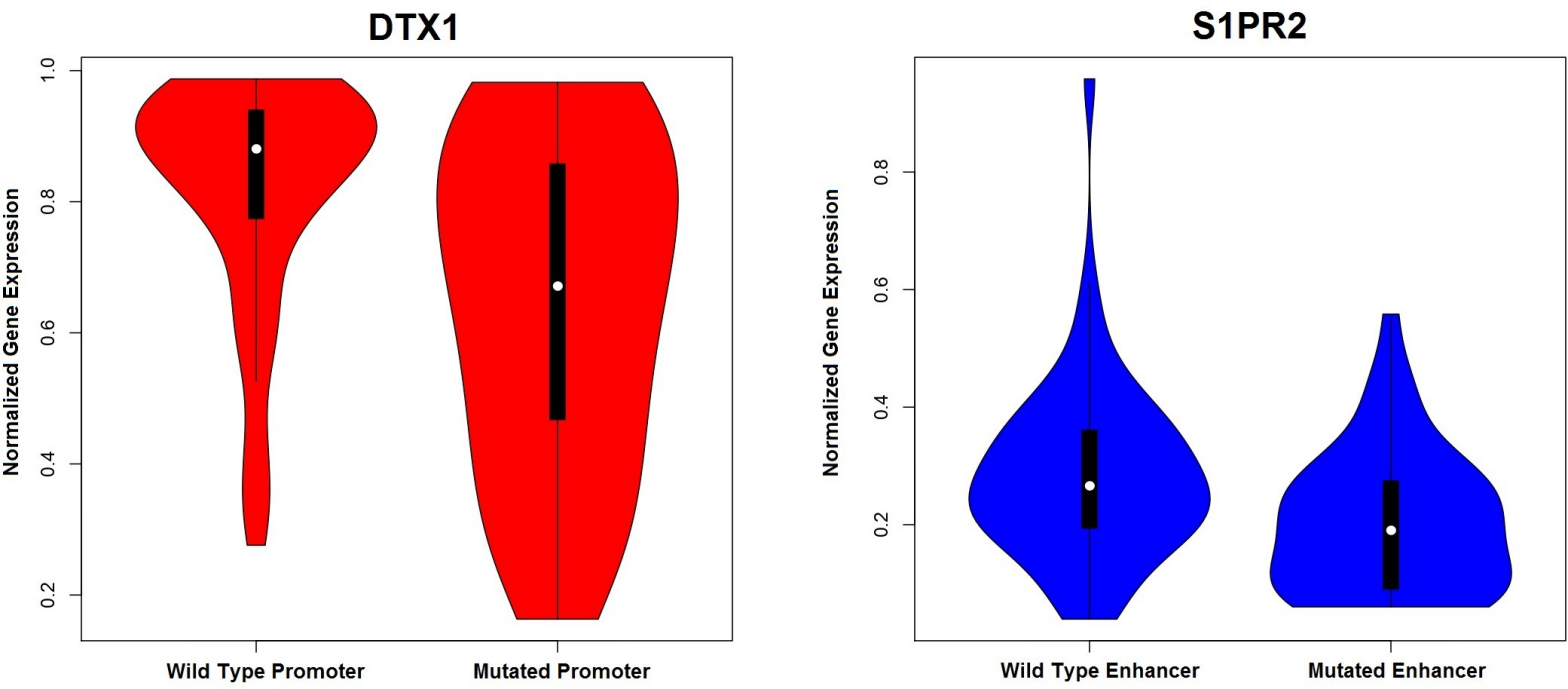
Violin plots representing the distribution of gene expression between mutated and unmutated *DTX1* promoters (left image) and *S1PR2* enhancers (right image).

A fraction of the patients (58%) had matched RNAseq data available. We tested association between regulatory mutations and expression of the adjacent genes. Strong underexpression of *DTX1* was associated with mutations in its promoter, which includes the *GH12J113056* enhancer region (promoter q-value, 2.90 x 10^−4^; enhancer q-value, 4.80 x 10^−3^, **Fig 4**). In the same line, mutations in *S1PR2* enhancer were also significantly associated with *S1PR2* underexpression (p-value 2.46 x 10^−4^, **Fig 4**). On the contrary, mutations in the *PAX5*/*ZCCHC7* enhancer were significantly associated with higher expression of *ZCCHC7* (q-value 3.31 x 10^−2^) but not with *PAX5* expression (q-value 0.93). Using non-linear kernel regression adjusted for diagnostic subtype, we could confirm independent associations for *DTX1* and *GH12J11305* mutations (p-value 2.51x 10^−3^) and *S1PR2* and its enhancer (p-value 5.01 x 10^−3^), but not for the association of *ZCCHC7* expression with mutations in the *PAX5/ZCCHC7* enhancer (p-value 0.17).

### 5. Genomic regions targeted by recurrent CNA

29 regions were significantly affected by focal CNA (FDR <10%), with a median affected region width of 1,327,586 bp (**S8 Table and S3** and **S4 Figs**). We detected 2 recurrently amplified loci and 27 recurrently deleted regions in 17 different chromosomes. Aside from immunoglobulin gene deletions, the most significant deletions were located in 13q14.2 (*DLEU1*, residual q-value 1.55 x 10^−14^), 3p12.3 (adjacent to *ROBO1*/*ROBO2* locus, q-value 2.90 x x10^-7^), 16q21 (*CDH5* and *CDH11* loci, residual q-value 1.06 x 10^−5^), 1p36.32 (*TNFRSF14*, residual q-value 7.56 x 10^−5^), 6q13 (*LINC00472* locus, residual q-value 8.93 x 10^−5^) and 13q33.3 (*TNFSF13B* locus, residual q-value 1.02 x 10^−4^). On the contrary, recurrent amplifications of the non-coding locus 9q34.11 (residual q-value 6.85 x 10^−3^) and the gene-rich region 13q31.3 (residual q-value 0.06) were identified. Other focal deletions affected genes involved in immune pathways (*IL5RA*) and oncogenic pathways, such as the tumor-suppressor *APAF1* (12q23.1), the immune check-point regulator *PVR* (19q13.31) and the cell cycle regulators *CDKN2A* and *CDKN2B* (9p21.3).

A fraction of the patients (58%) had matched RNAseq data available. Significant positive Pearson’s correlations between tumor/normal log2 rations and local gene expression were discovered in 16 CNAs (q-value <0.1; **S9 Table**). This included positive associations between losses in 1p36.32, 3p26.2, 4q35.2, 7p22.3 and 18q23 with the expression of the cancer-related genes *CDKN11B*, *CRBN*, *IRF2*, *PRKAR1B* and *NFATC*.

### 6. Differential events between B-cell lymphoma subtypes

We studied the distribution of the significant genomic events across the different B-cell lymphoproliferative subtypes (**S10 Table**). The greatest number of significant disparities (FDR < 5%) was discovered between CLL *versus* DLBCL and CLL *versus* follicular lymphoma. We detected 77 differential events between CLL and DLBCL, 34 differential events between CLL and follicular lymphoma, 11 differential events between CLL and Burkitt lymphoma, 17 differential events between DLBCL and Burkitt lymphoma, 11 differential events between follicular lymphoma and DLBCL and 9 differential events between follicular lymphoma and Burkitt lymphoma. Overall, 76.43% of all differential events were between CLL and any of the other lymphomas.

Although most differential events were less common in CLL, *IGH* deletions were highly enriched in CLL compared with the remaining lymphomas, and *IGL* deletions were more frequent in CLL compared to follicular lymphoma. Additionally, 11p15.5 deletions were more frequent in CLL than in follicular lymphoma or DLBCL, and 11q22.3 deletions were significantly more frequent in CLL than in DLBCL. In the same line, non-coding mutations in the *IGH* locus were significantly more frequent in CLL than in DLBCL or follicular lymphoma, and those in the *IGL* loci predominated in CLL over DLBCL. Furthermore, non-coding mutations in *RP11-789C2*.*1* (4q28.3) and in the first intron of *BACH2* (6q15) were significantly increased in CLL compared with DLBCL. On the contrary, coding mutations in 97 driver genes were significantly depleted in CLL compared with the remaining disease subgroups, likely reflecting the lower mutational burden on CLL. Additionally, 10 structural aberrations (9 deletions and 1 amplification) were significantly less frequent in CLL than in DLBCL or follicular lymphoma. The most significant were particularly predominant in DLBCL cases, and these were 6q26 loss (q-value 5.36 x 10^−5^), 16q21 loss (q-value 4.56 x 10^−4^) and 13q13.3 gain (q-value 4.56 x 10^−4^).

As expected, mutations in *MYC*, *ID3* and *CCND3* were more frequent in Burkitt Lymphoma than in DLBCL or follicular lymphoma, and additionally we also detected the significant enrichments of Burkitt Lymphomas in *TP53* and *FBXO11* mutations. On the contrary, mutations in KMT2D and BCL2 were more prevalent among follicular lymphoma and DLBCL than in Burkitt Lymphoma. 1p36.32 deletion was absent in Burkitt lymphoma, but noncoding mutations in *RP11-44H4*.*1* (3q27.3) were enriched in Burkitt lymphoma compared to DLBCL. Finally, the comparison of follicular lymphoma with DLBCL revealed significant differences in the mutational frequency of 11 genes. Of these, mutations in *CREBBP*, *KMT2D*, *TNFRSF14* and *RRAGC* were enriched in follicular lymphoma, and those of *BTG2*, *HLA-A*, *PIM1*, *IGLL5*, *SOCS1*, *CD83* and *SGK1* were enriched in DLBCL.

## Discussion

Different analyses of B-cell lymphoproliferative disorders have deconvoluted part of the complex genomic landscape of these neoplasms. Despite extensive evidence in other fields [20, 34], this is the first combined analysis of whole genomes of B-cell lymphoid tumors performed to our knowledge. In this work, we detected 112 recurrently mutated genes across the genomes of different B-cell lymphoid malignancies, of which 31 (27.7%) were not previously described in any of the analyzed tumor subtypes. Among these, some of the most frequently mutated (*FAM230A*, *FAM186A* and *PABPC3*) are barely characterized genes with testis-biased expression. On the contrary, many others play roles in pathways linked with lymphomagenesis. For example, *GRB2* and *INPP5D* participate in the B-cell receptor pathway [47, 48]; *LTB* and *TRAF6* regulate NFKB pathway activity [49, 50], and *ANGPT1* and *RPS6KA6* play a role in the MAPK pathway [51, 52]. Functional evidence supports the implication of the transcription factor *RFX7* [47–53] and the zinc finger protein *ZFP36L1* [54] in oncogenesis. Several other genes are members of the family of known lymphoma drivers, such as *CXCR5* [55], *HIST2H3D* [56], *ID2* [57] and *IRF1* [58]. Additionally, 180 regulatory regions were significantly enriched in mutations, with a significant contribution of aSHM target loci (67.7% of cases). Importantly, we could detect regulatory mutations accompanied by aberrant underexpression of the tumor suppressors *DTX1* [59, 60] and *S1PR2* [61, 62].

31 significantly mutated subnetworks involving 313 genes were discovered. In comparison with single gene approaches, this perspective provides a more complete landscape of mutational processes in B-cell lymphomas, and points towards the existence of new altered proteomic subnetworks in lymphomas. As a result, the genes *CREBBP*, *BCL2*, *EEF1A1*, *GNA13*, *STAT6* and *TP53*, and the pathways *“B-cell receptor”*, *“Apoptosis”*, *“Notch signalling”*, *“Polycomb Repressive Complex”* and *“Toll-like receptor”* emerge as master players of lymphomagenesis. Nevertheless, our results also support the implication of a myriad of novel players in the pathogenesis of B-cell lymphoid disorders, such as cell signalling proteins, cell-cycle regulators, ion transporters, cytoskeleton proteins, vesicle trafficking factors, extracellular enzymes and immunity genes. For some of these, the association with lymphomagenesis is well established, as in the case of *CR2*/*CD21* [63], *CD81* [64], *GLI1* [65], *SMO* [66] and the self-activating autocrine loops of *IL10* and *IL13* with their receptors [67, 68]. For other genes, likely associations can be inferred from its function, such as the cell-cycle checkpoint protein *MAD1L1* [69] and the angiogenesis inhibitor *BAI2* [70]. Finally, another group of genes belongs to emerging pathways in cancer whose function in B-cell lymphomas awaits further elucidation, such as glutamate receptors [71], ion channels [72] and microvesicles [73].

Focal recurrent structural alterations were detected in 29 loci. These events tended to affect known drivers of lymphomagenesis, and some were previously described, such as 13q14.2 deletions (*RB1* gene) [74], *CDKN2A*/*CDKN2B* [75], *TP58* [76], *DLEU1* (13q13.2) [8], and *ILR5RA* [77]. On the contrary, other novel deletions were either described in other tumors, such as *CDH11* in retinoblastoma [78] or *ROBO1* in breast cancer [79]; or affected genes vinculated with cancer pathways such as the tumor suppressor & non-coding RNA *LINC00472* [80], the B-cell specific maturation regulator *TNFRSF13C* (*BAFF receptor*) [81], the immune checkpoint *PVR* [82], the receptor tyrosine kinase-coupled signaling regulator *SIRPB1* [83] or the proapoptotic gene *APAF1* [84]. Furthermore, recurrent amplifications in a noncoding region within 9q34.11 were also detected, whose function needs to be further clarified.

Finally, some clues are provided about the distribution of these mutational events across B-cell tumor subtypes. A relative depletion of CLL in mutations affecting common drivers was found, in line with the lower mutational burden of this tumor. Additionally, several differences in mutation frequencies were also detected between follicular lymphoma, DLBCL and Burkitt lymphoma. As expected, mutations in frequent drivers such as *CREBBP*, *KMT2D*_,_ *TNFRSF14* and *RRAGC* were more frequent among follicular lymphomas, those of *MYC*, *CCND3* and *ID3* prevailed among Burkitt lymphoma and those of *BTG2*, *PIM1*, *SGK1* and *SOCS1* were more frequent among DLBCL. Some of these findings are concordant with reported frequencies of driver genes across distinct lymphoma subtypes [85–87], whereas others provide new clues about the pathogenesis and possible drug targets in these tumors. For example, the increased mutational burden of *TP53* and the BCL6-regulator gene *FBXO11* among Burkitt lymphomas suggest an increased deregulation of these pathways in this disease [88, 89]. Additionally, the skewed mutational profile of the immune check-point regulator *CD83* [90] towards DLBCL tumors might have both biological and therapeutic implications.

This work, as many others, has some limitations. For example, although the included B-cell disorders represent a majority of patients in real practice, other frequent B-cell malignancies need to be taken into account in the future. Furthermore, protein network analysis is still limited by incomplete annotation of the protein interactome and by the type of input scores that can be used as input. Additionally, it should be noted that data produced from different research groups can be affected by batch effects, which is the reason why we used a uniform and optimized pipeline for the analysis.

In conclusion, we present an integrated overview of the genomic drivers of some of the most frequent B-cell lymphoproliferative disorders. Our results shed new light about the pathogenic mutations and structural aberrations in coding and noncoding regulatory regions of the genome of B-cell lymphoproliferative disorders, and pinpoint towards disease-specific mutational events that might be useful both for therapeutic and diagnostic purposes.

## Supporting information

S1 Fig Volcano plot of the most significantly enriched KEGG terms in Network 1 genes. (PNG)

S2 Fig Volcano plot of the most significantly enriched KEGG terms in Network 2 genes. (PNG)

S3 Fig Deletion plot produced by Gistic2.0. (JPG)

S4 Fig Amplification plot produced by Gistic2.0. (JPG)

S1 Table Significantly mutated genes detected by dNdScv. (XLSX)

S2 Table Significantly mutated genes detected by MutSigCV. (XLSX)

S3 Table Significantly mutated genes detected by OncodriveFML. (XLSX)

S4 Table List of all significantly mutated driver genes. (XLSX)

S5 Table Significant subnetworks revealed by Hierarchical HotNet. (XLSX)

S6 Table Overrepresentation analysis of Network 1 and Network 2 genes in KEGG pathways terms. (XLSX)

S7 Table List of regulatory mutations significantly enriched in noncoding mutations (FDR <0.1). The last two columns indicate if the region maps to known target gene of aSHM, or if the mutational signature in that loci is consistent with aSHM.
(XLSX)

S8 Table List of significant and recurrent focal CNA regions (FDR <10%). (XLSX)

S9 Table List of significant positive correlations between tumor/normal log2 ratios of recurrent CNA and local gene expression. FDR is reported in case a CNA affected the locus of more than 1 gene.
(XLSX)

S10 Table Differential analysis of mutation events across the different subtypes of B-cell lymphoid neoplasms. Only events with FDR <5% are shown.
(XLSX)
