# Supplementary figures and images for: Detection of new drivers of frequent B-cell lymphoid neoplasms using an integrated analysis of whole genomes
Source: PLoS One. 2021 May 4;16(5):e0248886. doi: 10.1371/journal.pone.0248886 (PMC8096002; doi:10.1371/journal.pone.0248886)

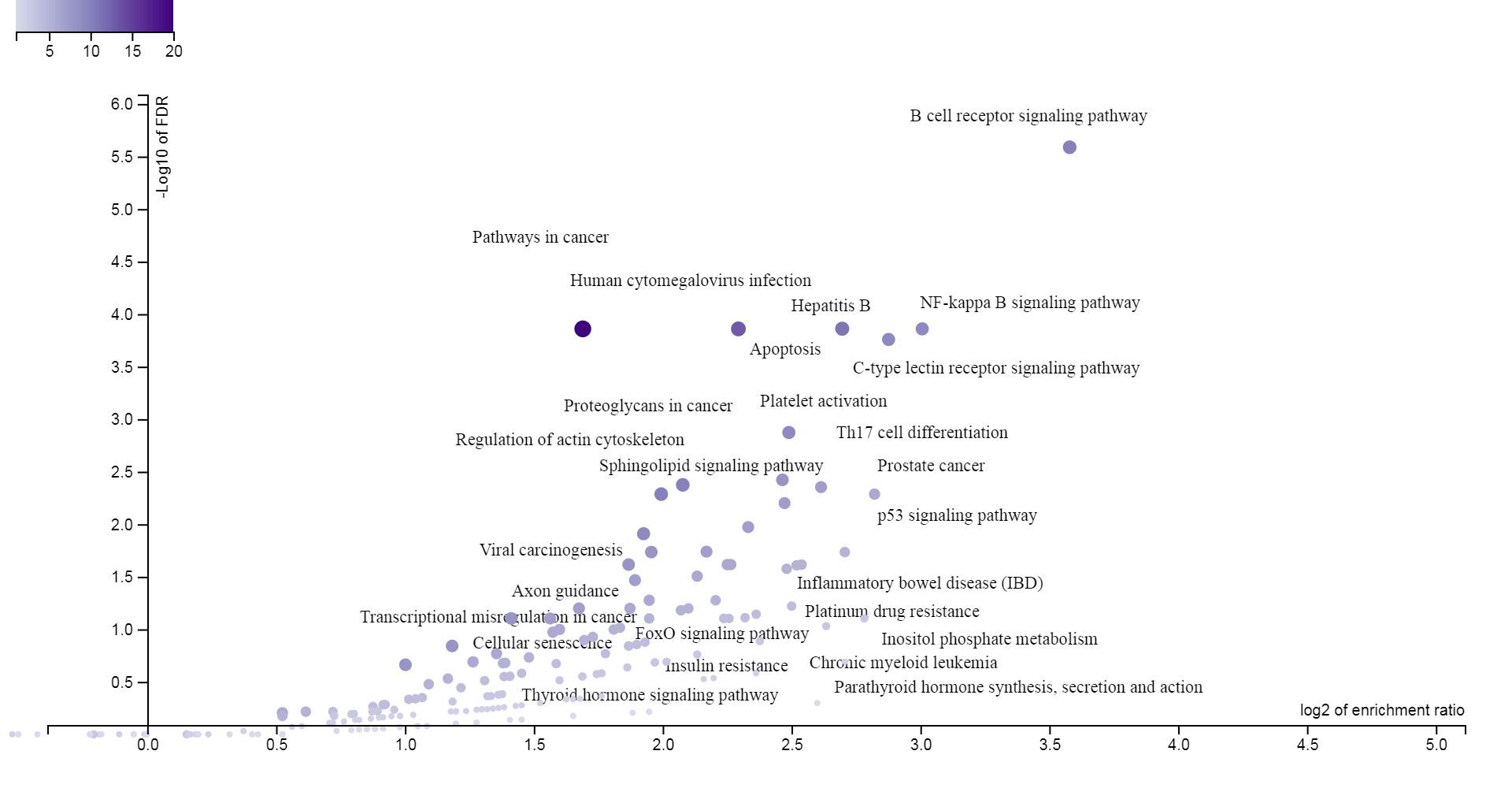

Supplement: S1 Fig — (PNG) [file pone.0248886.s001.png]

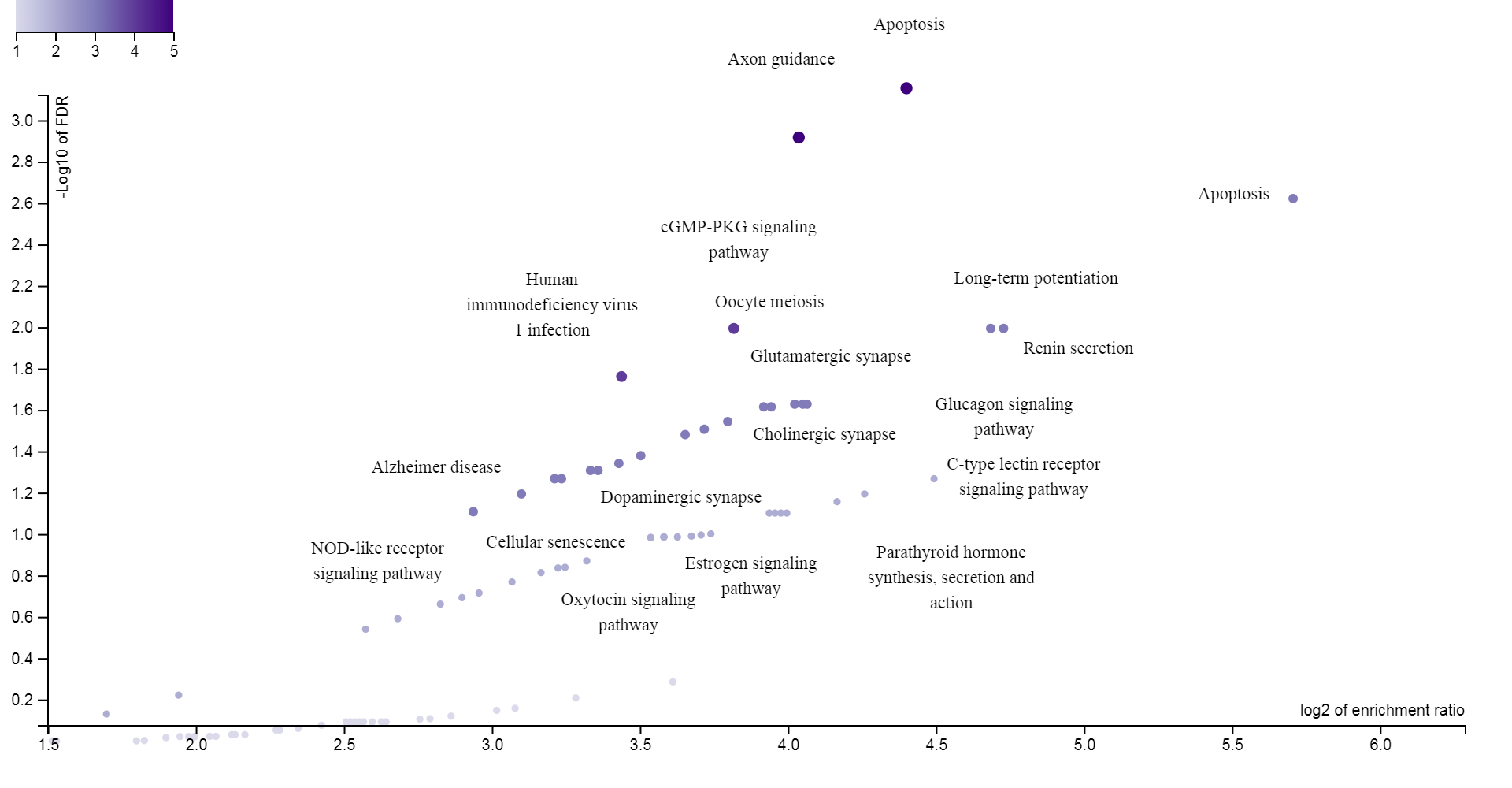

Supplement: S2 Fig — (PNG) [file pone.0248886.s002.png]

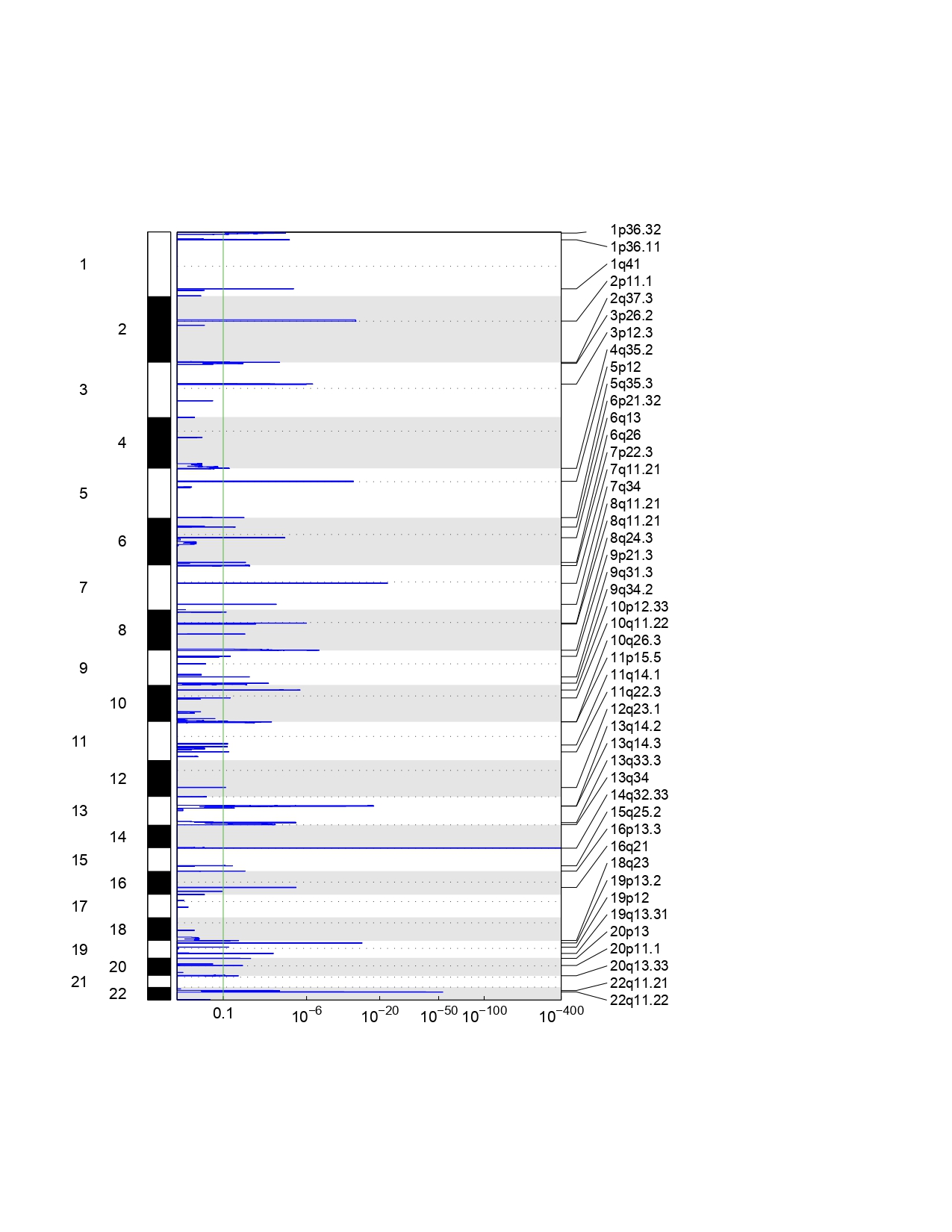

Supplement: S3 Fig — (JPG) [file pone.0248886.s003.jpg]

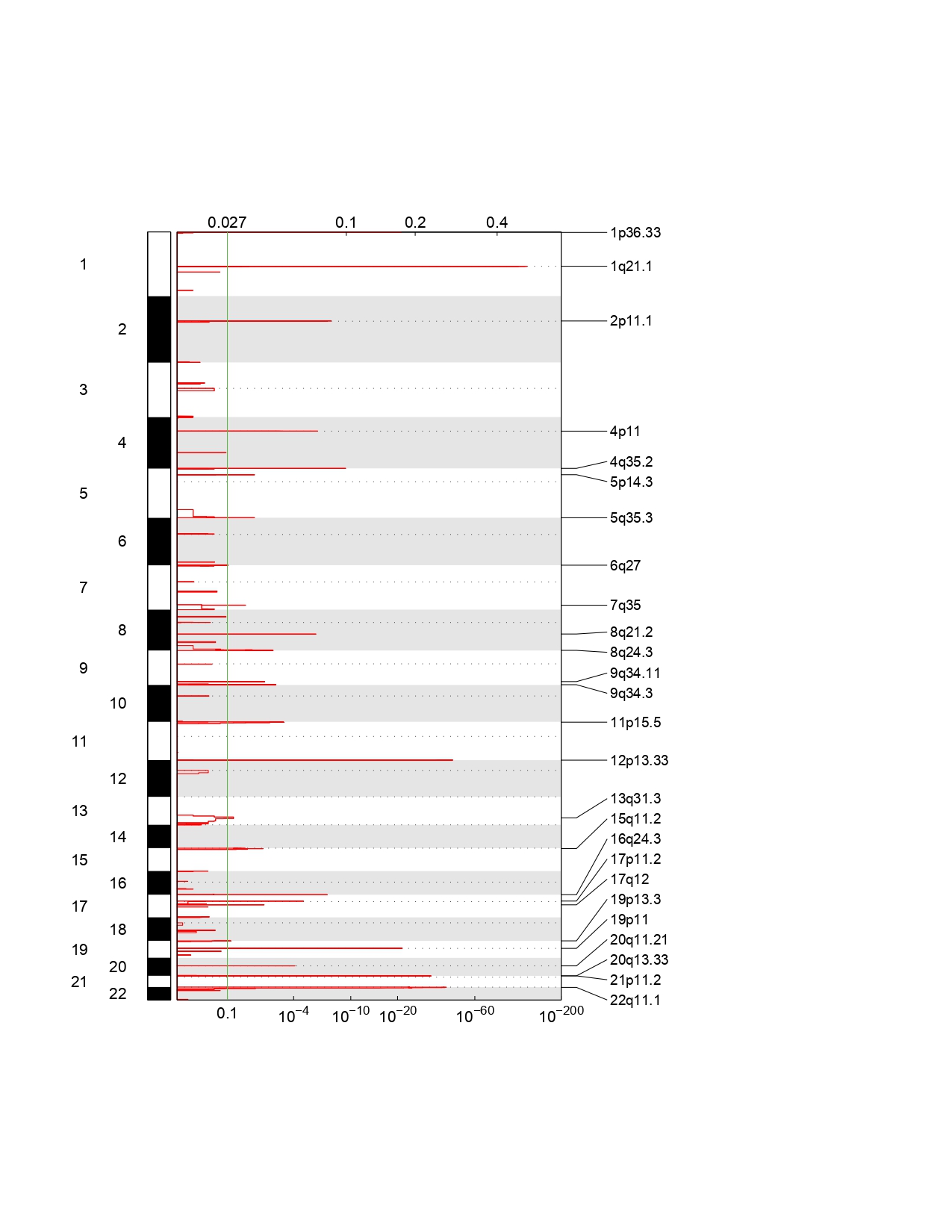

Supplement: S4 Fig — (JPG) [file pone.0248886.s004.jpg]
